# Determinants of self-reported correct knowledge about tuberculosis transmission among men and women in Malawi: evidence from a nationwide household survey

**DOI:** 10.1186/s12879-021-05836-y

**Published:** 2021-01-30

**Authors:** Peter A. M. Ntenda, Razak Mussa, Steve Gowelo, Alick Sixpence, Andy Bauleni, Atusayi Simbeye, Alfred Matengeni, Ernest Matola, Godfrey Banda, Christopher C. Stanley, Susan Banda, Owen Nkoka

**Affiliations:** 1 Malaria Alert Centre (MAC), College of Medicine (CoM), University of Malawi (UNIMA), Private Bag 360, Chichiri, Blantyre 3, Malawi; 2 Centre for Reproduction Health (CRH), College of Medicine (CoM), University of Malawi (UNIMA), Private Bag 360, Chichiri, Blantyre 3, Malawi; 3 School of Public Health and Family Medicine (SPHFM), College of Medicine (CoM), University of Malawi (UNIMA), Private Bag 360, Chichiri, Blantyre 3, Malawi; 4 School of Public Health (SPH), Taipei Medical University (TMU), No. 250, Wuxing Street, Xinyi District, Taipei City, 110 Taiwan; 5 Institute for Health Research and Communication (IHRC), P.O Box 1958, Lilongwe, Malawi; 6 Institute of Health and Wellbeing, University of Glasgow, Glasgow, UK

**Keywords:** Tuberculosis transmission, TB knowledge, Determinants, Malawi

## Abstract

**Background:**

Correct knowledge about transmission of tuberculosis (TB) can influence better health-seeking behaviors, and in turn, it can aid TB prevention in society. Therefore, this study aimed to examine the prevalence and predictors of self-reported correct knowledge about TB transmission among adults in Malawi.

**Methods:**

We conducted a secondary analysis of the data obtained from the Malawi Demographic and Health Survey, 2015/16 (MDHS 2015/16). Questions regarding self-reported TB transmission were computed to evaluate the correct knowledge about TB transmission. The factors associated with the correct knowledge about Tb were assessed using univariate and multivariable logistic regression.

**Results:**

Overall, the prevalence of correct knowledge about TB transmission in the general population of Malawian adults was 61.5%. Specifically, the prevalence of correct knowledge about TB transmission was 63.6 and 60.8% in men and women, respectively. Those aged 35–44 years, having secondary or high education, belonging to the richest household, being exposed to mass media, being in professional/technical/managerial, having knowledge that “TB can be cured”, and those living in urban areas were significantly associated with correct knowledge about TB transmission.

**Conclusions:**

The findings of this study show that if appropriate strategies for TB communication and education to address the rural masses, young individuals, poor individuals, and individuals in the agriculture sector are put it place, can enhance TB prevention in Malawi.

## Background

Tuberculosis (TB), an infectious disease which is caused by a bacteria called *Mycobacterium tuberculosis* (MTB), continues to be a major public health issue [1, 2]. Globally, approximately 10 million people were infected with TB in 2018, of which 1.5 million cases (including 251,000 people with human immunodeficiency virus – HIV) resulted in deaths, thus making it the world’s top infectious killer [3]. Furthermore, 44% of new TB cases occurred in the South-East Asian region, followed by the African region, with 24% of new cases and the Western Pacific with 18% [2, 4]. Unfortunately, over 90% of all TB cases and deaths occur in developing countries who have relatively fragile healthcare systems [5]. Additionally, like most African countries, TB remains a significant cause of morbidity and mortality in Malawi [6]. In 2018, Malawi had a TB incidence of 181 per 100,000 people, with HIV coinfection a counting for about 54% of the total cases [7].

It is known that having the correct knowledge about the symptoms and transmission mode of a disease is essential for disease prevention, screening, early detection, and early treatment-seeking behaviors – thereby improving overall management of health conditions [8–10]. Early diagnosis of TB among suspected individuals can prevent its transmission and eventually, reduce TB deaths [2]. However, studies have shown that inadequate knowledge about cause, mode of transmission and symptoms associated with TB are major barriers to prompt diagnosis and treatment of the disease [11–13]. Studies on TB have demonstrated that correct knowledge regarding transmission, clinical manifestations, and preventive and control methods of TB result in the declining incidence of TB yearly [14]. Generally, TB is highly contagious and most often affects the lungs (pulmonary TB), but can also affect other sites (extrapulmonary TB) [4]. The disease is transmitted from one person to another when people with pulmonary TB expel bacteria into the air (especially when coughing, sneezing or spitting) hence putting everybody in their immediate environment risk [2, 15]. Thus, knowledge of TB transmission is a fundamental basis for individuals taking protective measures to avoid becoming infected, or transmitting it to others for those with active disease [16].

The World Health Organization (WHO) declared TB as a global emergency in 1993 and later launched the Directly Observed Therapy short course (DOTs) strategy [17]. The DOTs therapeutic approach, adopted in many countries, including Malawi, depends on affected individual’s presenting at a health care facility rather than community case detection [18]. While Malawi’s National TB Control Program has been recognized internationally for its effective approach to TB control, the disease remains underdiagnosed in Malawi [19, 20]. In a cohort study investigating the prevalence of HIV and tuberculosis in adults with chronic cough in Malawi, it was found that nearly a third of all TB case in the cohort were not diagnosed previously [20]. Inadequate awareness of the disease was one of the main contributors to delayed diagnosing and treatment seeking [20]. In addition, another study reported that inadequate knowledge about cause and transmission of TB coupled with low self-awareness of personal risk to TB, cultural and traditional beliefs about sources of TB influenced delayed treatment seeking among adults aged 18 years and older from rural communities in Malawi [21]. Further, evidence shows that knowledge is an important predictor of health care seeking behavior and adherence to treatment [22, 23]. Hence developing effective strategies to improve knowledge about the disease and its transmission is one of the approach of accelerating progress towards Sustainable Development Goal (SDG) 3.3 that aims to end the tuberculosis epidemic by 2030 [24].

[25]. Studies from different settings have examined the predictors of self-reported correct knowledge about TB transmission and found that age [26, 27], gender [11, 26–28], education levels [26–29], household wealth [28], occupation [29], religion [11], exposure to mass media [11, 26, 30], geographical region [26–28], and perception that TB can be cured [11] were the most significant predictors. Understanding knowledge of TB in general and its determinants can inform policy developers and implementers of an effective community-based health promotion programs. Few studies have been conducted in Malawi to examine the knowledge about tuberculosis among different groups of the community [31–33]. However, none of these studies have estimated the predictors of TB related knowledge using nationally representative data.

Therefore, using the population-based data, this study aimed to examine the prevalence and predictors of self-reported correct knowledge about Tb transmission among adults in Malawi.

## Methods

### Data source, study design, sampling procedures, and data collection

The current study used data taken from the 2015–16 Malawi Demographic and Health Survey (MDHS). The 2015–16 MDHS sample was selected using a two-stage cluster sampling design and produced a nationally representative sample. The census sampling frame is considered as a complete list of all the census standard enumeration areas (SEAs). Thus, in the first stage, 850 SEAs (i.e., 173 SEAs in urban areas and 677 SEAs in rural areas) were selected with probability proportional to the SEA size. During the second stage, a fixed number of 30 and 33 households per urban rural cluster/SEA, respectively, were selected with an equal probability systematic selection criterion. All women and men of reproductive age 15–49 years and 15–54 years respectively, who were either permanent residence of the selected households or visitors who stayed in the household the night prior to the data collection were eligible for the interviews. The MDHS selected a total of 27,516 households, of which 24,562 women and 7478 men were successfully interviewed for the response rate of 97.7 and 94.6% respectively. Using women’s and men’s questionnaires, data were collected on socio-demographic characteristics and major health indicators, including knowledge, attitudes, and behaviors related to other health issues such as injections, smoking, fistula, tuberculosis HIV/ acquired immune deficiency syndrome (AIDS), and non-communicable diseases (NCDs). One of the key aims of The DHS Program is to collect data that are comparable across countries. Thus, to achieve this, standard model questionnaires have been developed and these model questionnaires—which have been reviewed and modified in each of the eight phases of The DHS program—form the basis for the questionnaires that are implemented in each country. The datasets for women and men were explored and after excluding respondents with missing data, a total of 28,862 respondents (6937 men and 21,925 women) were included in our analysis.

### Variables

#### Dependent variable

The dependent variable considered in this study was correct and adequate knowledge regarding the mode of TB transmission. This variable was created from the following 6 questions to evaluate the correct knowledge regarding mode of TB transmission among adult male and female.

i. TB is spread from person to person through the air when coughing or sneezing?
ii. TB can be transmitted by sharing utensils?
iii. TB can be transmitted through food?
iv. TB can be transmitted by touching a person with TB?
v. TB can be transmitted through sexual contact?
vi. TB can be transmitted through mosquito bites?

For the purposes of this study, the response to Q1 “Through air when coughing or sneezing” was used to measure the knowledge about the mode of TB transmission. The responses from ‘Q2’ to ‘Q6’ were regarded as misconceptions. However, individuals who responded ‘yes’ to the Q1 and responded ‘no’ to the other questions were recorded to have correct knowledge.

#### Independent variables

The present study considered the following covariates as independent variables; sex of the respondents, age of the respondents, educational level, wealth index, religion, occupation, marital status, amount of media exposure, perception about TB cure, perception about keeping secret when family member gets TB, place of residence, geographical religion, and ethnicity. These variables were selected after a thoroughly review of literature [11, 12, 34, 35]. The covariates were categorized as follows: sex of the respondents (male/ female), age of the respondents in years (< 25/ 25–34/ 35–44/≥45), educational level (no formal education/ primary/ secondary or high), wealth index (poorest/ poorer/ middle/ richer/ richest), religion (Roman catholic/ Church of Central African Presbyteria/ Anglican/ Seventh Day Adventist/ Baptist/ other Christian/ Muslim/ No religion/other), occupation (not working/ professional or technical or managerial/ clerical or sales or services/ agricultural employee/ skilled manual/ unskilled manual), marital status (never in union/ currently in union / formerly in union), amount of media exposure (0/ 1/ 2/ 3), Tuberculosis can be cured (no/ yes), keep secret when family member gets TB (no/ yes), place of residence (urban/ rural), geographical religion (northern/ central /southern), and ethnicity (Chewa/ Tumbuka/ Lomwe/ Tonga/ Yao/ Sena/ Nkhonde/ Ngoni/ Mang’anja/ Nyanja/ Other). In this study, exposure to mass media was derived from three items specifically television, newspaper, and radio. Amount of media exposure was constructed by quantifying the number of frequencies each media was attended to. The scores for amount of media exposure ranged from 0 (least possible score) to 3 (being the highest). Wealth index is defined as a composite measure of a household’s cumulative living standard and was created using easy-to-collect data on a household’s ownership of selected assets. These assets included televisions, bicycles, materials used for constructing the house, access to safe drinking water, sanitation facilities, and other characteristics of a household. Through a statistical technique named principal component analysis (PCA), placed the household into quintiles [36].

### Statistical analysis

We conducted our analyses while taking into account the complex design of the survey (i.e. weighting, clustering, and stratification). First, the baseline statistics were presented as frequency and weighted percentage. Second, the bivariate analyses using Chi-Square test were performed to explore the distribution of the selected characteristics according to the correct knowledge about the mode of TB transmission among adult women and men. Third, using the generalized estimating equation (GEE) logistic regression, the multivariable analyses were performed to investigate the strength of associations between the selected factors and correct knowledge about TB transmission. GEE models were used to account for the correlated responses within the hierarchical data such DHS data [37, 38]. Variables were selected for analysis based on their importance in literature [39]. We further selected variables that were significant (*p < 0.25*) in the Chi-Square test for adjustment in the GEE multivariable analyses so as to avoid large type II errors. Both unadjusted and adjusted odds ratios (aORs) and 95% confidence intervals (CIs) with their *p*-values were presented. *P-values* < 0.05 were considered as statistically significant. All analyses were performed using SAS software for Windows, version 9.4 (SAS Institute, Cary, NC, USA).

### Ethical considerations

The 2015–2016 MDHS was implemented by the National Statistical Office (NSO) and the Community Health Sciences Unit (CHSU). The protocols and procedures that were developed for data collection were reviewed and approved by the ICF Macro Institutional Review Board (ICF Macro IRB) and the Malawi National Health Science Research Committee. The MDHS 2015–2016 complied with all requirements of the US Department of Health and Human Services’ the 45 Code of Federal Regulations 46 (45 CFR 46), Protection of Human Subjects [40]. Before this study was conducted, the authors sought permission from the MEASURE DHS for use of the data beyond the primary purpose by which data were collected. At the beginning of each interview, informed consent (both written and oral consent) was obtained from all eligible participants. Furthermore, a parent or guardian provided consent prior to the participation by a child or anybody below the age of 18 years [40]. Overall, data obtained from respondents under the DHS program is anonymous as names are of the participants are not written down thus ethics approval for this study was not required.

## Results

### Baseline characteristics of the study participants by sex

A total of 28,862 adults (6937 males and 21,925 females) were sampled and analyzed in this study. Table 1 presents the baseline characteristics of the study participants stratified by sex. The overall prevalence of correct about TB knowledge in the general population of Malawi was adequate (61.5%). Most of the respondents (40.6%) were distributed in the age group 15 to 24 years and near two-thirds (59.2%) of the respondents had primary school education. Nearly 30.0% of respondents were residing in the richest households while about two-thirds (64.3%) currently in union. Approximately 40% of the participants did not have any form of mass media and a similar proportion (38.6%) of respondents were employed in agriculture sectors. Furthermore, over three-fourth (77.4%) of participants were rural dwellers and 44.98% were southern region dwellers.

**Table 1:** Descriptive statistics of Individual and community characteristics by sex, Malawi Demographic Health Survey, 2015–16

Variable	Male *n (%)* 6937 (24.04)	Female *n (%)* 21,925 (75.96)	Overall *n (%)* 28,862 (100.00)	*P*-value
Individual-level factors				
Age (years)				<.0001
< 25	2838 (40.91)	8891 (40.55)	11,729 (40.64)	
25–34	1896 (27.33)	7012 (31.98)	8908 (30.86)	
35–44	1441 (20.77)	4626 (21.10)	6067 (21.02)	
≥ 45	762 (10.98)	1396 (6.37)	2158 (7.48)	
Educational level				<.0001
No education	366 (5.28)	2285 (10.42)	2651 (9.19)	
Primary	3834 (55.27)	13,260 (60.48)	17,094 (59.23)	
Secondary or high	2737 (39.46)	6380 (29.10)	9117 (31.59)	
Wealth index^†^				<.0001
Poorest	939 (13.54)	3631 (16.56)	4570 (15.83)	
Poorer	1204 (17.36)	3824 (17.44)	5028 (17.42)	
Middle	1328 (19.14)	3982 (18.16)	5310 (18.40)	
Richer	1467 (21.15)	4440 (20.25)	5907 (20.47)	
Richest	1999 (28.82)	6048 (27.58)	8047 (27.88)	
Religion				<.0001
Roman catholic	1299 (18.73)	3916 (17.86)	5215 (18.07)	
CCAP	1180 (17.01)	3590 (16.37)	4770 (16.53)	
Anglican	361 (5.20)	1133 (5.17)	1494 (5.18)	
Seventh Day Adventist/Baptist	491 (7.08)	1679 (7.66)	2170 (7.52)	
Other Christian	2776 (40.02)	9215 (42.03)	11,991 (41.55)	
Muslim	655 (9.44)	2273 (10.37)	2928 (10.14)	
No religion/other	175 (2.52)	119 (0.54)	294 (1.02)	
Marital status				<.0001
Never in union	2575 (37.12)	4526 (20.64)	7101 (24.60)	
Currently in union	4108 (59.22)	14,443 (65.87)	14,443 (64.27)	
Formerly in union	254 (3.66)	2956 (13.48)	2956 (11.12)	
Amount of media exposure^‡^				<.0001
0	1076 (15.51)	9046 (41.26)	10,122 (35.07)	
1	2101 (30.29)	6684 (30.49)	8785 (30.44)	
2	2157 (31.09)	3988 (18.19)	6145 (21.29)	
3	1603 (23.11)	2207 (10.07)	3810 (13.20)	
Occupation				<.0001
Not working	960 (13.84)	7148 (32.60)	8108 (28.09)	
Professional/technical/managerial	490 (7.06)	1280 (5.84)	1770 (6.11)	
Clerical/sales/services	478 (6.89)	1537 (7.01)	2015 (6.98)	
Agricultural employee	2701 (38.94)	8448 (38.53)	11,149 (38.63)	
Skilled manual	800 (11.53)	335 (1.53)	1135 (3.93)	
Unskilled manual	1508 (21.74)	3177 (14.49)	4685 (16.23)	
Tb can be cured				<.0001
No	1010 (14.56)	4375 (19.95)	5385 (18.66)	
Yes	5927 (85.44)	17,550 (80.05)	23,477 (81.34)	
Community-level factors				
Place of residence				0.3626
Urban	1594 (22.98)	4923 (22.45)	6517 (22.58)	
Rural	5343 (77.02)	17,002 (77.55)	22,345 (77.42)	
Geographical region				0.0025
Northern	1447 (20.86)	4286 (19.55)	5733 (19.86)	
Central	2489 (35.88)	7657 (34.92)	10,146 (35.15)	
Southern	3001 (43.26)	9982 (45.53)	12,983 (44.98)	
Ethnicity				<.0001
Chewa	2167 (31.24)	6556 (29.90)	8723 (30.22)	
Tumbuka	747 (10.77)	2326 (10.61)	3073 (10.65)	
Lomwe	1251 (18.03)	4128 (18.83)	5379 (18.64)	
Tonga	253 (3.65)	871 (3.97)	1124 (3.89)	
Yao	704 (10.15)	2337 (10.66)	3041 (10.54)	
Sena	299 (4.31)	949 (4.33)	1248 (4.32)	
Nkhonde	121 (1.74)	290 (1.32)	411 (1.42)	
Ngoni	878 (12.66)	2829 (12.90)	3707 (12.84)	
Mang’anja	178 (2.57)	499 (2.28)	677 (2.35)	
Nyanja	115 (1.66)	491 (2.24)	606 (2.10)	
Other	224 (3.23)	649 (2.96)	873 (3.02)	
Correct knowledge of TB transmission				<.0001
No	2522 (36.36)	8602 (39.23)	11,124 (38.54)	
Yes	4415 (63.64)	13,323 (60.77)	17,738 (61.46)	

*TB* tuberculosis, *OR* Odds Ratio, *AOR* adjusted Odds Ratio, *CI* Confidence Interval

^‡^Frequency of reading newspaper or magazine, Frequency of listening to radio and frequency of watching television

^†^Composite measure of a household’s cumulative living standard

### Prevalence of self-reported tuberculosis knowledge about TB transmission

**Fig. 1:**
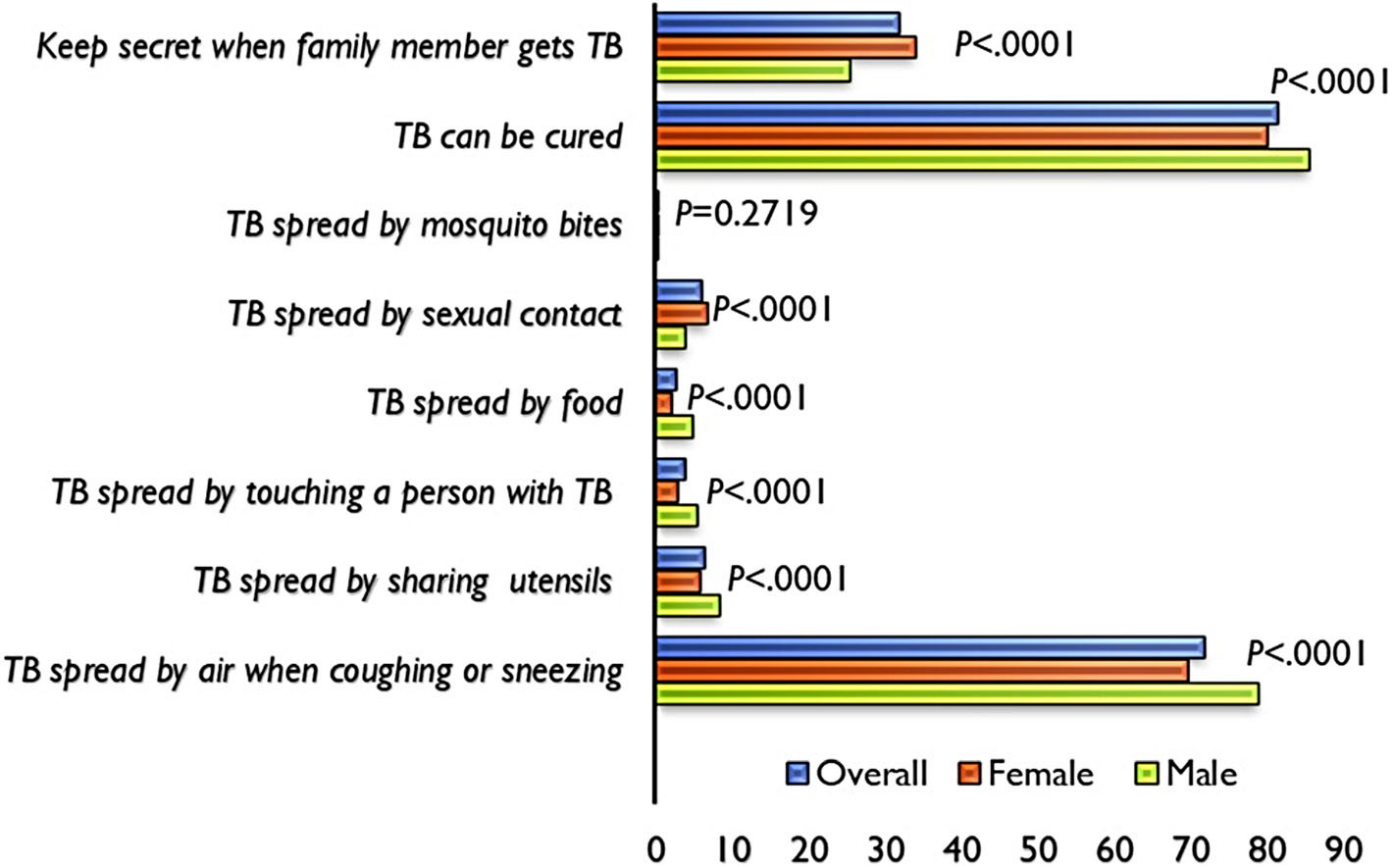
Domains used to measure correct knowledge about tuberculosis transmission in Malawi

Table 2 presents the domains that were used to measure correct knowledge about the mode of TB transmission among women and men of reproductive age. Ninety-seven percent of men and 94% of women had heard of TB. Overall, 8908 (71.83%) of the participants responded correctly that TB is spread from one person to another through air by coughing or sneezing. Furthermore, 81.34% of respondents believed that TB can be cured and 31.89% of participants would want a family member’s TB status kept secret. There were significant differences between men and women who reported having heard of TB, such that 69.64% of women and 78.77% of men reported that TB is spread through the air by coughing or sneezing (*P* < .0001). Additionally, 85.44% of women and 80.05% of men believe that TB can be cured (*P* < .0001) while 25.39% of men and 33.94% of women would want a family member’s TB status kept secret (*P* < .0001). Figure 1 shows the distribution of the domain that were used to construct knowledge related to TB transmission.

**Table 2:** Proportion of respondents with correct knowledge about tuberculosis transmission in Malawi, MDHS 2015–16

Variable	Male n (%) 6937 (24.04)	Female n (%) 21,925 (75.96)	Overall n (%) 28,862 (100.00)	*P*-value
TB spread by air when coughing or sneezing (Yes)	5464 (78.77)	15,268 (69.64)	8908 (71.83)	<.0001
TB spread by sharing utensils (No)	6365 (91.75)	20,664 (94.25)	27,029 (93.65)	<.0001
TB spread by touching a person with TB (No)	6556 (94.51)	21,314 (97.21)	27,870 (96.56)	<.0001
TB spread by food (No)	6609 (95.27)	21,485 (97.99)	28,094 (97.34)	<.0001
TB spread by sexual contact (No)	6670 (96.15)	20,458 (93.31)	27,128 (93.99)	<.0001
TB spread by mosquito bites (No)	6917 (99.71)	21,842 (99.62)	28,759 (99.64)	0.2719
TB can be cured (Yes)	5927 (85.44)	17,550 (80.05)	23,477 (81.34)	<.0001

*TB* Tuberculosis

### Prevalence of correct knowledge about TB transmission by selected characteristics

Table 3 shows the prevalence of correct knowledge about TB transmission among men and women of reproductive age by sociodemographic characteristics. The prevalence of correct knowledge about TB transmission was significant different from those who had incorrect knowledge by sex of the respondents (*P* < .0001), age of the respondents (*P* < .0001), educational level (*P* < .0001), household wealth (*P* < .0001), religion (*P* < .0001), amount of media exposure (*P* < .0001), respondent’s occupation (*P* < .0001), respondents with a belief that TB can be cured (*P* < .0001), respondents would want a family member’s TB status kept secret (*P* < .0001), place of residence (*P* < .0001), geographical region (*P* < .0001), and ethnicity (*P* < .0001).

**Table 3:** Prevalence of correct knowledge of TB transmission by individual and community characteristics MDHS 2015–16

Variable	Over all n (%) 28,862 (100.00)	No n (%) 11,124 (38.54)	Yes n (%) 17,738 (61.46)	*P*-value
Individual-level factors				
Sex				<.0001
Male	6937 (24.46)	2522 (22.67)	4415 (24.89)	
Female	21,925 (75.96)	8602 (77.33)	13,323 (75.11)	
Age (years)				<.0001
< 25	11,729 (40.64)	4869 (43.77)	6860 (38.67)	
25–34	8908 (30.86)	3202 (28.78)	5706 (32.17)	
35–44	6067 (21.02)	2220 (19.96)	3847 (21.69)	
≥ 45	2158 (7.48)	833 (7.49)	1325 (7.47)	
Educational level				<.0001
No education	2651 (9.19)	1238 (11.13)	1413 (7.97)	
Primary	17,094 (59.23)	7348 (66.06)	9746 (54.94)	
Secondary or high	9117 (31.59)	2538 (22.82)	6579 (37.09)	
Wealth index^†^				<.0001
Poorest	4570 (15.83)	2076 (18.66)	2494 (14.06)	
Poorer	5028 (17.42)	2140 (19.24)	2888 (16.28)	
Middle	5310 (18.40)	2154 (19.36)	3156 (17.79)	
Richer	5907 (20.47)	2308 (20.75)	3599 (20.29)	
Richest	8047 (27.88)	2446 (21.99)	5601 (31.58)	
Religion				<.0001
Roman catholic	5215 (18.07)	2020 (18.16)	3195 (18.01)	
CCAP	4770 (16.53)	1680 (15.10)	3090 (17.42)	
Anglican	1494 (5.18)	564 (5.07)	930 (5.24)	
Seventh Day Adventist/Baptist	2170 (7.52)	716 (6.44)	1454 (8.20)	
Other Christian	11,991 (41.55)	4946 (44.44)	7048 (39.73)	
Muslim	2928 (10.14)	1081 (9.72)	1874 (10.41)	
No religion/other	294 (1.02)	120 (1.08)	174 (0.98)	
Marital status				0.5732
Never in union	7101 (24.60)	2755 (24.77)	4346 (24.50)	
Currently in union	14,443 (64.27)	7158 (64.35)	11,393 (64.23)	
Formerly in union	2956 (11.12)	1211 (10.89)	1999 (11.27)	
Amount of media exposure^‡^				<.0001
0	10,122 (35.07)	4291 (38.57)	5831 (32.87)	
1	8785 (30.44)	3563 (32.03)	5222 (29.44)	
2	6145 (21.29)	2215 (19.91)	3930 (22.16)	
3	3810 (13.20)	1055 (9.48)	2755 (15.53)	
Occupation				<.0001
Not working	8108 (28.09)	3094 (27.81)	5014 (28.27)	
Professional/technical/managerial	1770 (6.11)	401 (3.60)	1369 (7.72)	
Clerical/sales/services	2015 (6.98)	720 (6.47)	1295 (7.30)	
Agricultural employee	11,149 (38.63)	4799 (43.14)	6350 (35.80)	
Skilled manual	1135 (3.93)	384 (3.45)	751 (4.23)	
Unskilled manual	4685 (16.23)	1726 (15.52)	2959 (16.68)	
Tuberculosis can be cured				<.0001
No	5385 (18.66)	2927 (26.31)	2458 (13.86)	
Yes	23,477 (81.34)	8197 (73.69)	15,280 (86.14)	
Community-level factors				
Place of residence				<.0001
Urban	6517 (22.58)	1925 (17.30)	4592 (25.89)	
Rural	22,345 (77.42)	9199 (82.70)	13,146 (74.11)	
Geographical region				<.0001
Northern	5733 (19.86)	2671 (24.01)	3062 (17.26)	
Central	10,146 (35.15)	4031 (36.24)	6115 (34.47)	
Southern	12,983 (44.98)	4422 (39.74)	8561 (48.26)	
Ethnicity				<.0001
Chewa	8723 (30.22)	3558 (31.98)	5165 (29.12)	
Tumbuka	3073 (10.65)	1361 (12.23)	1712 (9.65)	
Lomwe	5379 (18.64)	1704 (15.32)	3675 (20.72)	
Tonga	1124 (3.89)	554 (4.98)	570 (3.21)	
Yao	3041 (10.54)	1080 (9.71)	1961 (11.06)	
Sena	1248 (4.32)	461 (9.71)	787 (4.44)	
Nkhonde	411 (1.42)	170 (1.53)	241 (1.36)	
Ngoni	3707 (12.84)	1353 (12.16)	2354 (13.27)	
Mang’anja	677 (2.35)	240 (2.16)	437 (2.46)	
Nyanja	606 (2.10)	243 (2.18)	363 (2.05)	
Other	873 (3.02)	400 (3.60)	473 (2.67)	

*TB* tuberculosis, *OR* Odds Ratio, *AOR* adjusted Odds Ratio, *CI* Confidence Interval

^‡^Frequency of reading newspaper or magazine, Frequency of listening to radio and frequency of watching television

^†^Composite measure of a household’s cumulative living standard

### Factors associated with correct knowledge about TB transmission

Table 4 shows results of univariate and multivariate logistics regression that were used to test independent predictors of the given variables and assess their strength of associations between those predictors with correct knowledge about TB transmission. In univariate analysis characteristics that were significantly associated with correct knowledge about TB transmission included sex of the respondents, age of the respondents, educational level, household wealth, religion, amount of media exposure, occupation, tuberculosis can be cured, TB status kept secret, place of residence, geographical region, and ethnicity (all *P-*values less than 0.05). The results of multivariate logistics regression showed that respondents of age groups 25–34 years odds (adjusted odds ratio [aOR]: 1.219; 95% confidence interval [CI]: 1.145–1.297; *P* < .0001), 35–44 years (aOR: 1.275; 95% CI: 1.187–1.371; *P* < .0001), and 45+ years (aOR: 1.239; 95% CI: 1.116–1.375; *P* < .0001) had increased compared to those respondents of age group 15 to 24 years. The odds of having correct knowledge about TB transmission was also high in respondents with primary education (aOR: 1.253; 95% CI: 1.144–1.371; *P* < .0001) and secondary and high education (aOR: 2.000; 95% CI: 1.793–2.232; *P* < .0001) compared to those with no formal education. Furthermore, respondents from middle household wealth (aOR: 1.106; 95% CI: 1.016–1.205; *P- =* 0.0205), richer households (aOR: 1.096; 95% CI: 1.005–1.196; *P =* 0.0387), and richest households (aOR: 1.166; 95% CI: 1.053–1.292; *P =* 0.032) had increased odds of having correct knowledge about TB transmission compared to respondents from poorest households. Additionally, respondents who had exposure to 3 forms of mass media (aOR: 1.190; 95% CI: 1.076–1.315; *P =* 0.0007), respondents who believed that TB can be cured (aOR: 1.708; 95% CI: 1.600–1.823; *P* < .0001), respondents from urban (aOR: 1.165; 95% CI: 1.063–1.277; *P =* 0.0011), and respondents from Lomwe tribe (aOR: 1.286; 95% CI: 1.066–1.551; *P =* 0.0086) had also increased odds of having correct knowledge about TB transmission. Conversely, agricultural employee (aOR: 0.909; 95% CI: 0.843–0.981; *P* = 0.0143), respondents who would want a family member’s TB status to be kept secret (aOR: 0.886; 95% CI: 0839–0.935; *P* < .0001) had reduced odds of having correct knowledge about TB transmission. Furthermore, respondents from central region (aOR: 0.896; 95% CI: 0.819–0.980; *P =* 0.0161) and northern region (aOR: 0.581; 95% CI: 0.512–0.659; *P* < .0001), had also reduced odds of having correct knowledge about TB transmission compared to those from the southern region.

**Table 4:** Determinants of correct and adequate knowledge about tuberculosis transmission among adult men and women

Variable	Univariate		Multivariable	
	CrOR 95% (CI)	P-value	AOR 95% (CI)	*P*-value
Individual-level factors				
Sex				
Male	1.150 (1.085–1.218)	<.0001	1.012 (0.949–1.079)	0.7118
Female	1.000		1.000	
Age (years)				
< 25	1.000		1.000	
25–34	1.279 (1.206–1.357)	<.0001	1.219 (1.145–1.297)	<.0001
35–44	1.256 (1.175–1.342)	<.0001	1.275 (1.187–1.371)	<.0001
≥ 45	1.173 (1.064–1.293)	0.0014	1.239 (1.116–1.375)	<.0001
Educational level				
No education	1.000		1.000	
Primary	1.181 (1.083–1.286)	0.0002	1.253 (1.144–1.371)	<.0001
Secondary or high	2.225 (2.022–2.447)	<.0001	2.000 (1.793–2.232)	<.0001
Wealth index^†^				
Poorest	1.000		1.000	
Poorer	1.140 (1.048–1.240)	0.0022	1.081 (0.993–1.176)	0.0734
Middle	1.238 (1.139–1.346)	<.0001	1.106 (1.016–1.205)	0.0205
Richer	1.324 (1.218–1.439)	<.0001	1.096 (1.005–1.196)	0.0387
Richest	1.822 (1.670–1.987)	<.0001	1.166 (1.053–1.292)	0.0032
Religion				
Roman catholic	1.063 (0.828–1.364)	0.6319	0.957 (0.743–1.232)	0.7339
CCAP	1.212 (0.944–1.557)	0.1322	1.037 (0.804–1.338)	0.7768
Anglican	1.110 (0.845–1.459)	0.4523	1.145 (0.868–1.510)	0.3365
Seventh Day Adventist/Baptist	1.305 (1.006–1.693)	0.0452	1.073 (0.824–1.398)	0.6004
Other Christian	0.964 (0.754–1.231)	0.7675	0.951 (0.742–1.219)	0.6936
Muslim	1.042 (0.804–1.352)	0.7541	1.020 (0.777–1.339)	0.8855
No religion/other	1.000		1.000	
Amount of media exposure^‡^				
0	1.000		1.000	
1	1.103 (1.038–1.172)	0.0015	1.010 (0.948–1.076)	0.7571
2	1.306 (1.218–1.399)	<.0001	1.056 (0.978–1.140)	0.1621
3	1.787 (1.636–1.959)	<.0001	1.190 (1.076–1.315)	0.0007
Occupation				
Not working	0.934 (0.864–1.010)	0.0885	1.011 (0.931–1.097)	0.7992
Professional/technical/managerial	1.911 (1.676–2.180)	<.0001	1.336 (1.166–1.531)	<.0001
Clerical/sales/services	1.028 (0.917–1.153)	0.6346	0.893 (0.795–1.004)	0.0588
Agricultural employee	0.841 (0.780–0.907)	<.0001	0.909 (0.843–0.981)	0.0143
Skilled manual	1.125 (0.976–1.296)	0.1032	0.983 (0.850–1.137)	0.8176
Unskilled manual	1.000		1.000	
Tuberculosis can be cured				
No	1.000		1.000	
Yes	2.088 (1.960–2.223)	<.0001	1.708 (1.600–1.823)	<.0001
Community-level factors				
Place of residence				
Urban	1.674 (1.528–1.835)	<.0001	1.165 (1.063–1.277)	0.0011
Rural	1.000		1.000	
Geographical region				
Northern	0.588 (0.533–0.650)	<.0001	0.581 (0.512–0.659)	<.0001
Central	0.777 (0.715–0.845)	<.0001	0.896 (0.819–0.980)	0.0161
Southern	1.000		1.000	
Ethnicity				
Chewa	1.214 (1.025–1.439)	0.0249	1.052 (0.878–1.259)	0.5836
Tumbuka	1.125 (0.941–1.345)	0.1956	1.054 (0.886–1.253)	0.5553
Lomwe	1.709 (1.434–2.036)	<.0001	1.286 (1.066–1.551)	0.0086
Tonga	0.919 (0.738–1.145)	0.4520	0.898 (0.727–1.108)	0.3158
Yao	1.423 (1.184–1.710)	0.0002	1.122 (0.911–1.382)	0.2769
Sena	1.387 (1.120–1.719)	0.0028	1.100 (0.881–1.373)	0.4018
Nkhonde	1.199 (0.914–1.572)	0.1900	1.146 (0.878–1.497)	0.3156
Ngoni	1.398 (1.168–1.673)	0.0003	1.063 (0.882–1.281)	0.5220
Mang’anja	1.484 (1.173–1.877)	0.0010	1.086 (0.850–1.386)	0.5110
Nyanja	1.251 (0.979–1.599)	0.0735	0.997 (0.780–1.274)	0.9807
Other	1.000		1.000	

*TB* tuberculosis, *CrOR* Crude Odds Ratio, *AOR* adjusted Odds Ratio, *CI* Confidence Interval

^‡^Frequency of reading newspaper or magazine, Frequency of listening to radio and frequency of watching television

^†^Composite measure of a household’s cumulative living standard

## Discussion

The purpose of the current study was to examine the prevalence and factors associated with the correct knowledge concerning TB transmission among adults in Malawi. An understanding of such knowledge and its predictors is of great essence as it may help TB control programme managers and policymakers to develop effective community based health promotion programs [41]. Researchers have reported that individuals with lower levels of health knowledge are generally 1.5 to 3 times more likely to have poor health outcomes than their counterparts with higher literacy or health-related knowledge levels [42, 43]. Specifically, inadequate and poor health knowledge has been consistently associated with overall (1) individual’s poor health status, (2) lack of engagement with health care providers, (3) decreased comprehension of medical information, (4) lack of knowledge about medical conditions and related care, (5) increased mortality, (6) poor use of preventive health services, (7) poor self-reported health, and (8) increased rate and longer stay of hospitalizations [43, 44]. The present study showed that most of the participants had correct knowledge regarding TB transmission. Additionally, this study found that being in the age group 35–44 years, having secondary or high education, being in the richest household, exposure to all the three mode of mass media, being in professional/technical/managerial, having knowledge that TB can be cured and being urban dwellers were significantly associated with correct knowledge about TB transmission.

As with previous research [45], this study found that an increase in age was significantly associated with self-reported correct knowledge about TB transmission among adults in Malawi. Specifically, individual of age ≥ 25 years and above had more correct knowledge about TB transmission. Similarly, another study reported that correct answers to questions concerning TB were associated with increasing age [46]. Generally, the reason behind the TB knowledge getting increased with advanced age, possibly may be that older individuals might have had developed more correct attitudes and behaviors regarding the disease [45, 47].

We found also that respondents who had secondary or high education had higher likelihood of having correct knowledge about TB transmission. Our results are consistent with prior literature [48–50]. The explanation to this result might be that the highly educated individuals have great likelihood of having access to different sources of information and easily understand more complex messages [50–52]. Eventually, improved levels of education in the community can improve cognitive level and increase the general knowledge about infection control including TB and the general health of the people [53]. Furthermore, as with previous literature [26, 35], the current study found that respondents from the richest households had high chances of having correct knowledge about TB transmission. Generally, the an explanation to this finding might be that, people from households with better income, usually tend to have higher probability of acquiring improved health knowledge and better health seeking behavior [54, 55].

In agreement with previous studies [26, 30, 35], this study found that respondents who had exposed to television, radio and newspaper, being in professional/technical/managerial, having knowledge that TB can be cured and being urban dwellers were associated with correct knowledge about TB transmission. It is reported that, the media form a crucial role in patients’ knowledge about TB and, therefore, underscores the requisite for TB health education programmes among disadvantaged households [25, 56]. Furthermore, these findings suggest that respondents from urban areas may be more exposed for messages or information such as mass media and other health-related messages (e.g. television, ratio, newspapers, posters or billboards, peer educators, etc.) than women from rural areas who usually come from low socioeconomic backgrounds [25, 35]. Similarly, respondents with white collar jobs, might have easy access to health knowledge due to the nature of the education and work [57] and in turn may have the knowledge and they can develop positive attitude that TB can be cured. The observed variation in terms of TB knowledge among ethnic groups underscore the need for qualitative studies to understand this phenomenon.

### Strengths and limitations

This study cannot determine the causation between the exposure variables and the outcome variable due to the cross-sectional nature of the study design. Considering that the sample size of females was 3 times that of male participants, and that a large percentage of the participants were under the age of 25, the external validity of the results to the entire Malawian population maybe compromised (i.e., the results may not be generalizable to the Malawian population). Nevertheless, the factors observed in this study may help inform TB control programs aimed at improving overall TB knowledge in Malawi. However, despite these limitations, the findings presented in this study would contribute to our understanding of the determinants of TB transmission which may improve the quality of TB management in Malawi. These results can be generalized only in a certain specific population such as women and those age less than 25 years.

## Conclusion

The findings of this study revealed that if appropriate strategies for TB communication and education to address the rural masses, young individuals, poor individuals, and individuals in the agriculture sector are put it place, can enhance TB prevention in Malawi.

## Data Availability

The datasets generated and/or analyzed during the present study are available in The DHS Program repository. https://dhsprogram.com/data/dataset/Malawi_Standard-DHS_2015.cfm?flag=1

## Abbreviations

AIDS: Acquired immune deficiency syndrome
aORs: Adjusted odds ratios
CFR: Code of Federal Regulations
CHSU: Community Health Sciences Unit
Cis: Confidence intervals
DOTs: Directly Observed Therapy short course
GEE: Generalized estimating eq.
HIV: Human immunodeficiency virus
MDHS: Malawi Demographic and Health Survey
MTB: *Mycobacterium tuberculosis*
NCDs: Non-communicable diseases
NSO: National Statistical Office
PCA: Principal component analysis
SDG: Sustainable Development Goal
SEAs: Standard enumeration areas
TB: Tuberculosis
WHO: World Health Organization

## References

[1] World Health Organization (WHO). Global Tuberculosis Report 2020. Geneva: WHO; 2020.

[2] World Health Organization (WHO) (2019). Global tuberculosis report 2019.

[3] World Health Organization (WHO). Tuberculosis, Key facts. Geneva: WHO; 2020.

[4] World Health Organization (WHO) (2018). Global tuberculosis report 2018.

[5] Zaman K (2010). Tuberculosis: a global health problem. J Health Popul Nutr.

[6] United States Agency for International Development (USAID). Malawi Tuberculosis Fact Sheet. Malawi: USAID; 2016.

[7] Trading Econiomics (2020). Malawi - incidence of tuberculosis (per 100,000 people).

[8] Lemon SM, Hamburg MA, Sparling PF, Choffnes ER, Mack A. Global infectious disease surveillance and detection: assessing the challenges. Workshop summary. In Global infectious disease surveillance and detection: assessing the challenges. Workshop summary. National Academies Press; 2007. 21391348

[9] Khan A, Shaikh BT, Baig MA (2020). Knowledge, awareness, and health-seeking behaviour regarding tuberculosis in a Rural District of Khyber Pakhtunkhwa, Pakistan. Biomed Res Int.

[10] Williams G, Alarcon E, Jittimanee S, Walusimbi M, Sebek M, Berga E (2007). Best practice for the care of patients with tuberculosis: a guide for low-income countries.

[11] Sreeramareddy CT, Kumar HNH, Arokiasamy JT (2013). Prevalence of self-reported tuberculosis, knowledge about tuberculosis transmission and its determinants among adults in India: results from a nation-wide cross-sectional household survey. BMC Infect Dis.

[12] Luba TR, Tang S, Liu Q, Gebremedhin SA, Kisasi MD, Feng Z (2019). Knowledge, attitude and associated factors towards tuberculosis in Lesotho: a population based study. BMC Infect Dis.

[13] Obuku EA, Meynell C, Kiboss-Kyeyune J, Blankley S, Atuhairwe C, Nabankema E (2012). Socio-demographic determinants and prevalence of tuberculosis knowledge in three slum populations of Uganda. BMC Public Health.

[14] Zhang T, Liu X, Bromley H, Tang S (2007). Perceptions of tuberculosis and health seeking behaviour in rural Inner Mongolia, China. Health Policy (New York).

[15] World Health Organization (WHO). Global tuberculosis control. Geneva: WHO; 2011.

[16] Murray EJ, Bond VA, Marais BJ, Godfrey-Faussett P, Ayles HM, Beyers N (2013). High levels of vulnerability and anticipated stigma reduce the impetus for tuberculosis diagnosis in Cape Town, South Africa. Health Policy Plan.

[17] World Health Organization (WHO). WHO Global Tuberculosis Programme. A global emergency, WHO report on the TB epidemic. Geneva: WHO; 1994.

[18] Kumwenda M, Desmond N, Hart G, Choko A, Chipungu GA, Nyirenda D (2016). Treatment-seeking for tuberculosis-suggestive symptoms: a reflection on the role of human agency in the context of universal health coverage in Malawi. PLoS One.

[19] Kanyerere H, Harries AD, Tayler-Smith K, Jahn A, Zachariah R, Chimbwandira FM (2016). The rise and fall of tuberculosis in Malawi: associations with HIV infection and antiretroviral therapy. Trop Med Int Heal.

[20] Nliwasa M, MacPherson P, Mukaka M, Mdolo A, Mwapasa M, Kaswaswa K (2016). High mortality and prevalence of HIV and tuberculosis in adults with chronic cough in Malawi: a cohort study. Int J Tuberc Lung Dis.

[21] Nyasulu P, Phiri F, Sikwese S, Chirwa T, Singini I, Banda HT (2016). Factors influencing delayed health care seeking among pulmonary tuberculosis suspects in rural communities in Ntcheu District, Malawi. Qual Health Res.

[22] Finnie RKC, Khoza LB, van den Borne B, Mabunda T, Abotchie P, Mullen PD (2011). Factors associated with patient and health care system delay in diagnosis and treatment for TB in sub-Saharan African countries with high burdens of TB and HIV. Trop Med Int Heal.

[23] Warsi SMA, Danish SH, Ahmad F, Khan AI, Khan MP, Bano S (2016). Tuberculosis knowledge and health seeking behaviour: a tale of two districts of Sindh, Pakistan. J Pak Med Assoc.

[24] Floyd K, Glaziou P, Houben R, Sumner T, White RG, Raviglione M (2018). Global tuberculosis targets and milestones set for 2016–2035: definition and rationale. Int J Tuberc Lung Dis.

[25] Mushtaq MU, Majrooh MA, Ahmad W, Rizwan M, Luqman MQ, Aslam MJ (2010). Knowledge, attitudes and practices regarding tuberculosis in two districts of Punjab, Pakistan. Int J Tuberc Lung Dis.

[26] Pengpid S, Peltzer K (2019). Knowledge, attitudes, and practices regarding tuberculosis in Timor-Leste: results from the demographic and health survey 2016. J Prev Med Public Heal.

[27] Mondal MNI, Nazrul HM, Chowdhury MRK, Howard J (2014). Socio-demographic factors affecting knowledge level of tuberculosis patients in Rajshahi City, Bangladesh. Afr Health Sci.

[28] Datiko DG, Habte D, Jerene D, Suarez P. Knowledge, attitudes, and practices related to TB among the general population of Ethiopia: findings from a national cross-sectional survey. PLoS One. 2019;14. 10.1371/journal.pone.0224196 PMC6816561 31658300

[29] Hossain S, Zaman K, Quaiyum A, Banu S, Husain A, Islam A (2015). Factors associated with poor knowledge among adults on tuberculosis in Bangladesh: results from a nationwide survey. J Health Popul Nutr.

[30] de Freitas IM, Popolin MP, Touso MM, Yamamura M, Rodrigues LBB, Santos Neto M (2015). Factors associated with knowledge about tuberculosis and attitudes of relatives of patients with the disease in Ribeirão Preto, São Paulo, Brazil. Rev Bras Epidemiol.

[31] Chizimba R, Christofides N, Chirwa T, Singini I, Ozumba C, Sikwese S (2015). The association between multiple sources of information and risk perceptions of tuberculosis, Ntcheu District, Malawi. PLoS One.

[32] Nyasulu P, Kambale S, Chirwa T, Umanah T, Singini I, Sikwese S (2016). Knowledge and perception about tuberculosis among children attending primary school in Ntcheu District, Malawi. J Multidiscip Healthc.

[33] Nyasulu P, Sikwese S, Chirwa T, Makanjee C, Mmanga M, Babalola JO (2018). Knowledge, beliefs, and perceptions of tuberculosis among community members in Ntcheu district, Malawi. J Multidiscip Healthc.

[34] Portero Navio JL, Rubio Yuste M, Pasicatan M (2002). Socio-economic determinants of knowledge and attitudes about tuberculosis among the general population of metro Manila, Philippines. Int J Tuberc Lung Dis.

[35] Agho KE, Hall J, Ewald B (2014). Determinants of the knowledge of and attitude towards tuberculosis in Nigeria. J Health Popul Nutr.

[36] Rutstein SO, Johnson K (2004). The DHS wealth index. DHS comparative reports no. 6. Calvert ORC Macro.

[37] Hardin JW. Generalized estimating equations (GEE). Encycl Stat Behav Sci. 2005.

[38] Ballinger GA (2004). Using generalized estimating equations for longitudinal data analysis. Organ Res Methods.

[39] Maldonado G, Greenland S (1993). Simulation study of confounder-selection strategies. Am J Epidemiol.

[40] The DHS Program. What-We-Do > Protecting the Privacy of DHS Survey Respondents. Meas DHS+, ORC Macro n.d.

[41] Kigozi NG, Heunis JC, Engelbrecht MC, Janse van Rensburg AP, van Rensburg HCJD. Tuberculosis knowledge, attitudes and practices of patients at primary health care facilities in a South African metropolitan: research towards improved health education. BMC Public Health 2017;17:795. doi:10.1186/s12889-017-4825-3. 10.1186/s12889-017-4825-3 PMC5633895 29017526

[42] DeWalt DA, Berkman ND, Sheridan S, Lohr KN, Pignone MP (2004). Literacy and health outcomes. J Gen Intern Med.

[43] Protheroe J, Nutbeam D, Rowlands G (2009). Health literacy: a necessity for increasing participation in health care.

[44] Jayasinghe UW, Harris MF, Parker SM, Litt J, van Driel M, Mazza D (2016). The impact of health literacy and life style risk factors on health-related quality of life of Australian patients. Health Qual Life Outcomes.

[45] Ou Y, Luo Z, Mou J, Ming H, Wang X, Yan S (2018). Knowledge and determinants regarding tuberculosis among medical students in Hunan, China: a cross-sectional study. BMC Public Health.

[46] Montagna MT, Napoli C, Tafuri S, Agodi A, Auxilia F, Casini B (2014). Knowledge about tuberculosis among undergraduate health care students in 15 Italian universities: a cross-sectional study. BMC Public Health.

[47] Butler M, Talley KMC, Burns R, Ripley A, Rothman A, Johnson P (2011). Values of older adults related to primary and secondary prevention.

[48] Hassan AO, Olukolade R, Ogbuji QC, Afolabi S, Okwuonye LC, Kusimo OC, et al. Knowledge about tuberculosis: a precursor to effective TB control—findings from a follow-up national KAP study on tuberculosis among Nigerians. Tuberc Res Treat. 2017;2017. 10.1155/2017/6309092 PMC5624149 29075531

[49] Huddart S, Bossuroy T, Pons V, Baral S, Pai M, Delavallade C (2018). Knowledge about tuberculosis and infection prevention behavior: a nine city longitudinal study from India. PLoS One.

[50] Kusheno FT, Nguse TM, Gebretekle GB. Assessment of knowledge and attitude of tuberculosis patients in direct observation therapy program towards multidrug-resistant tuberculosis in Addis Ababa, Ethiopia: a cross-sectional study. Tuberc Res Treat. 2020;2020. 10.1155/2020/6475286 PMC7298339 32566290

[51] Gill HK, Gill N, Young SD (2013). Online technologies for health information and education: a literature review. J Consum Health Internet.

[52] Ntenda PAM, Chuang K-Y, Tiruneh FN, Chuang Y-C (2017). Analysis of the effects of individual and community level factors on childhood immunization in Malawi. Vaccine.

[53] Wang M, Han X, Fang H, Xu C, Lin X, Xia S, et al. Impact of health education on knowledge and behaviors toward infectious diseases among students in Gansu Province, China. Biomed Res Int. 2018;2018. 10.1155/2018/6397340 PMC5863350 29707573

[54] Abadura SA, Lerebo WT, Kulkarni U, Mekonnen ZA (2015). Individual and community level determinants of childhood full immunization in Ethiopia: a multilevel analysis. BMC Public Health.

[55] Case A, Lubotsky D, Paxson C (2002). Economic status and health in childhood: the origins of the gradient. Am Econ Rev.

[56] Hashim DS, Al Kubaisy W, Al Dulayme A (2003). Knowledge, attitudes and practices survey among health care workers and tuberculosis patients in Iraq. EMHJ-Eastern Mediterr Heal J.

[57] Vandenplas Y, Basrowi RW, Sulistomo AW, Adi NP, Widyahening IS (2019). Breastfeeding Knowledge, Attitude, and Practice among White-Collar and Blue-Collar Workers in Indonesia.

